# Comparative analysis of postoperative outcomes following surgical and transcatheter edge-to-edge mitral valve repair for secondary mitral regurgitation: a meta-analysis & systematic review

**DOI:** 10.3389/fsurg.2025.1645272

**Published:** 2025-10-09

**Authors:** Dudy A. Hanafy, Adrian R. Sudirman, Sari Rahmawati, Hendry R. Satria, Safitri, Stefanus Nursalim, Muhammad R. Bachmid, Dwi G. Fardhani, Tri W. Soetisna, Sugisman

**Affiliations:** 1 Department of Cardiac, Thoracic, and Vascular Surgery, National Cardiovascular Center Harapan Kita, Jakarta, Indonesia; 2 Department of Cardiac, Thoracic, and Vascular Surgery, Faculty of Medicine, Universitas Indonesia, Jakarta, Indonesia

**Keywords:** mitral regurgitation, secondary MR, transcatheter, surgical, mitral valve repair

## Abstract

**Introduction:**

Mitral regurgitation (MR) affects approximately millions of people globally, predominantly older adults, leading to 0.88 million DALY and 34,000 deaths in 2019. Both ESC and ACC/AHA guidelines recommend intervention either surgery or transcatheter for secondary MR despite optimal medical therapy. The comparative effectiveness of SMVr vs. TEER for managing secondary MR remains uncertain, prompting a systematic review to assess outcomes, safety, and long-term implications.

**Method:**

This systematic review and meta-analysis were carried out and documented according to the PRISMA 2020 guidelines. Searches were conducted in the Embase, EBSCOHost, Medline, Sage, Science Direct, and Scopus databases.

**Result:**

This meta-analysis included eight studies and 6224 patients. Both SMVr and TEER showed similar rate of in-hospital mortality (3.85% vs. 2.83%, RR = 2.54; 95% CI = 0.59–10.95; *p* = 0.21; I^2^ = 57%), while SMVr was associated with a significantly lower incidence of post-discharge residual MR compared to TEER (RR = 0.27; 95% CI = 0.16–0.45; *p* < 0.01; I^2^ = 0%). However, SMVr showed a higher incidence of neurologic events, including stroke or TIA (1.89% vs. 0.94%, RR = 1.88; 95% CI = 1.16–3.05; *p* = 0.001; I^2^ = 0%). The rates of acute renal failure (5.26% vs. 5.29%, RR = 1.23; 95% CI = 0.84–1.80; *p* = 0.28; I^2^ = 9%) and postoperative myocardial infarction (1.91% vs. 1.81%, RR = 1.07; 95% CI = 0.71–1.62; *p* = 0.73; I^2^ = 0%) were higher in the SMVr group, but this was statistically insignificant. Mid-term mortality analysis favored SMVr over TEER, with lower mortality rates observed in SMVr patients (Rate Ratio 0.74; 95% CI, 0.63–0.88; *p* < 0.001; I²=27%), lower reintervention rates (RR = 0.29, *p* < 0.001), lower incidence rate ratio of recurrent MR (Rate Ratio = 0.56; 95% CI = 0.40–0.78; *p* = 0.0005; I2 = 0%) and heart failure rehospitalization (Rate Ratio = 0.81; 95% CI = 0.68–0.97; *p* = 0.02; I2 = 5%). SMVr patients were more likely to experience improvement in functional status (NYHA) compared to TEER patients (RR = 1.14, *p* < 0.006).

**Conclusion:**

SMVr has demonstrated better mid-term outcomes than TEER, including lower mortality rates, fewer reinterventions and rehospitalization, and improved functional status in patients with mitral regurgitation.

**Systematic Review Registration:**

identifier [CRD42024538771].

## 1 Introduction

Mitral regurgitation (MR) stands as the third most prevalent type of valvular heart disease, impacting roughly 24.2 million individuals globally. Given that MR predominantly affects older adults, it led to approximately 0.88 million disability-adjusted life-years (DALY) and 34,000 fatalities in 2019 (1).

According to the 2021 European Society of Cardiology (ESC) guidelines, valve surgery or intervention is strongly recommended (Class 1 recommendation) for patients with severe secondary mitral regurgitation who continue to experience symptoms despite receiving guideline-directed medical therapy (GDMT), which may include cardiac resynchronization therapy (CRT) if deemed appropriate or for those undergoing coronary artery bypass grafting (CABG) or other cardiac surgery. For patients with concomitant coronary artery or other cardiac disease requiring intervention who are not suitable for surgery, percutaneous coronary intervention (PCI), possibly followed by transcatheter edge-to-edge repair (TEER), should be considered (Class 2a recommendation). The decision regarding valve surgery or intervention should be made collaboratively by a structured Heart Team (2).

The 2020 ACC/AHA guidelines suggest (Class 2a recommendation) that transcatheter edge-to-edge mitral valve repair (TEER) may be considered appropriate for patients meeting specific criteria: suitable anatomy as determined by transesophageal echocardiography (TEE), left ventricular ejection fraction (LVEF) ranging from 20%–50%, left ventricular end-systolic dimension (LVESD) less than or equal to 70 mm, and pulmonary artery systolic pressure below or equal to 70 mm Hg. This recommendation applies to individuals with chronic severe secondary mitral regurgitation (MR) associated with impaired left ventricular systolic function (LVEF less than 50%) who continue to experience symptoms (NYHA class II, III, or IV) despite optimal guideline-directed medical therapy (GDMT) for heart failure (Stage D). For patients with severe secondary MR (Stages C and D), undergoing mitral valve surgery is considered reasonable, particularly when CABG is performed for the treatment of myocardial ischemia (Class 2b recommendation) (3).

However, as many as 50% of individuals with severe secondary MR do not undergo surgical intervention due to the significant procedural risks involved (4–7). In response, TEER has emerged as an alternative strategy for patients who are deemed unsuitable candidates for surgery due to contraindications or high surgical risk, provided they meet appropriate anatomical criteria. TEER represents a relatively low-risk option to alleviate symptoms and promote reverse left ventricular (LV) remodeling (8, 9). Nonetheless, it is frequently accompanied by residual and recurrent MR (8). While TEER has demonstrated superior short-term outcomes, it is noteworthy that it also exhibited significantly high major bleeding rates and higher medium-term major adverse cardiovascular events (MACE) compared to SMVr (10, 11).

There is still uncertainty regarding which interventional approach, SMVr or TEER, is superior in managing secondary mitral regurgitation.

This systematic review and meta-analysis aim to provide a comprehensive assessment of the outcomes following surgical vs. transcatheter edge-to-edge mitral valve repair, shedding light on their respective efficacy, safety, and long-term prognostic implications. By synthesizing existing evidence from relevant studies, this analysis seeks to inform clinical decision-making and contribute to the ongoing refinement of treatment strategies for mitral valve disease.

## 2 Materials and methods

This systematic review and meta-analysis was conducted and reported following the Preferred Reporting Items for Systematic Reviews and Meta-Analyses (PRISMA) 2020 guidelines (12).

### 2.1 Literature search

Embase, EBSCOHost, Medline, Sage, Science Direct, and Scopus were systematically searched from database inception through March 3, 2024, to identify studies comparing SMVr and TEER for treating secondary MR. No language or publication-year restrictions were applied. A search method was employed by a query of PubMed as follows: ((((mitral valve insufficiency[MeSH Terms]) AND (((secondary) OR (functional)) OR (ischemic))) AND (((((transcatheter) OR (percutaneous)) OR (edge-to-edge)) OR (mitraclip)) OR (mitraclips))) AND ((((open) OR (surgery)) OR (surgical)) OR (repair))) AND (((((((((mortality) OR (assessment, outcomes[MeSH Terms])) OR (Reintervention)) OR (rehospitalization)) OR (neurologic event)) OR (acute renal failure)) OR (Arrhythmia)) OR (length of stay)) OR (residual mitral regurgitation)). The adjusted keywords were then implemented in search strategies for other databases.

### 2.2 Study selection

A total of six reviewers (A.R.S, S.S, R.S., M.R.B., S.F., and D.G.F.) independently conducted the study selection process based on previously developed criteria. Discussions took place to resolve any disagreements between the reviewers. Titles and abstracts were used to evaluate studies at the initial stage of the study selection process; the full text was examined in subsequent stages. Selection criteria were based on the following criteria: (1) enrolled patients with symptomatic secondary or functional mitral regurgitation (MR), (2) comparison of postoperative outcomes between SMVr and TEER, (3) age 18 years or older above, and (4) RCT or cohort study. In SMVr group, concomitant procedures—such as tricuspid valve surgery and coronary artery bypass grafting (CABG)—were performed when indicated. Likewise, in the TEER group, relevant concomitant procedures, including percutaneous coronary interventions (PCI), were permitted. Patients were excluded from the study if they had primary or degenerative mitral regurgitation. The most appropriate article was selected if redundant publications with overlapping data were found.

### 2.3 Data extraction

Two reviewers (R.S. and S.S.) extracted demographic and study characteristics independently. Likewise, three reviewers (A.R.S, M.R.B., and D.G.F.) extracted study outcome data. Disagreements between reviewers were resolved by discussion. The resulting data is divided into short-term data and medium-term data. Short-term outcomes included in-hospital mortality, neurologic events, myocardial infarction, acute renal failure, residual MR after discharge, and length of stay. Long-term outcomes included reintervention rates, mortality during follow-up, recurrence of MR during follow-up, improved functional outcomes, and rehospitalization due to heart failure. Residual MR was defined as greater than moderate MR (>2+) on echocardiography at discharge, and recurrent MR was defined as MR greater than moderate (>2+) during follow-up after initial procedural success.

### 2.4 Quality assessment

The quality of the study was independently assessed by four reviewers (D.H., R.S., S.S., and A.R.S.) Any disagreements during the review process were settled through discussion. This study used the Newcastle-Ottawa Quality Assessment Scale (NOS) for cohort studies, which consists of eight questions graded from zero to nine and assesses three assessment elements: selection, comparability, and outcome. A methodological quality score of six or more was considered high quality, three to five was considered fair, and less than three was regarded as low quality (13).

### 2.5 Statistical analysis

The data was retrieved and recorded utilizing Microsoft Excel. Parametric continuous outcomes were reported as mean ± standard deviation (SD) or median with interquartile range (IQR), as appropriate. Categorical outcomes were reported as a proportion (percentage) of the total number of participants. To evaluate the impact of surgical technique and study heterogeneity, meta-analyses were conducted. The Mantel–Haenszel (MH) method was used to estimate pooled risk ratios (RRs) and 95% confidence interval (CI) for dichotomous outcomes. For time-to-event outcomes, incidence rate ratios were used to account for differences in follow-up times between studies. Pooled estimates were presented graphically using forest plots. Statistical heterogeneity was assessed using the I^2^ statistic and the *χ*^2^ test. I^2^ values of 25%, 50%, and 75% were considered to indicate low, moderate, and high heterogeneity, respectively. A random-effects model was applied when heterogeneity was significant (I² > 50% or *p* < 0.10), while a fixed-effect model was used otherwise. All meta-analyses were conducted utilizing the “meta” package in R (version 4.5.1) for analyses of proportions. Incidence rate ratios and risk ratios were calculated using RevMan version 5.4.

This study has been registered and publicly available in PROSPERO with identifier number CRD42024538771.

## 3 Result

### 3.1 Study selection

**Figure 1:**
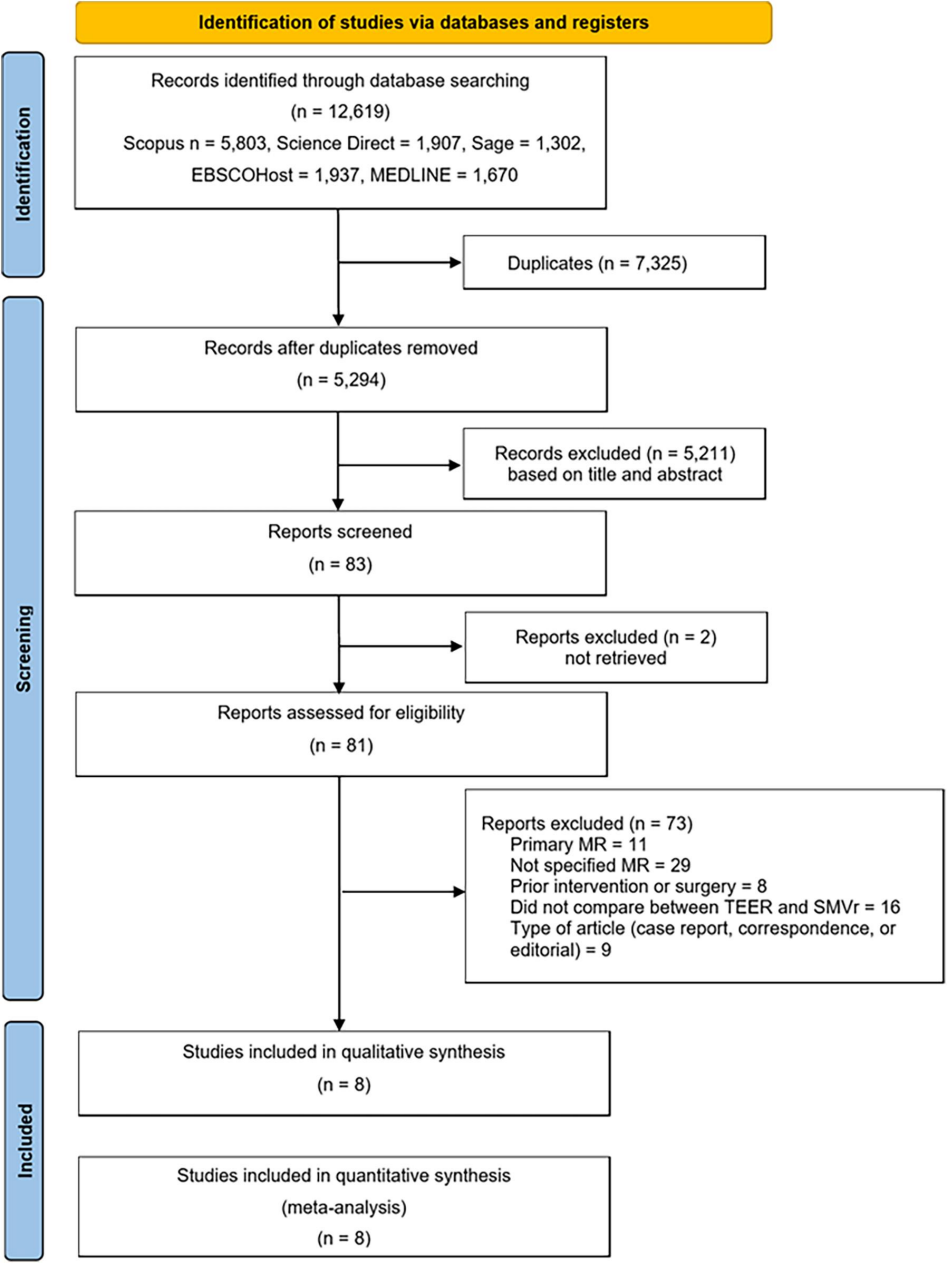
Study selection process with PRISMA flowchart.

The literature search results obtained from Scopus, Science Direct, Sage, EBSCOhost, and MEDLINE, are illustrated in Figure 1. In total, 12,619 articles were retrieved from the search. Out of the 83 articles that underwent purview extraction and subsequent analysis to meet the research criteria, two were not found in full text. Case reports or correspondence comprised nine articles, subjects with no specific type of MR comprised twenty-nine articles, subjects with primary MR comprised eleven articles, subjects with prior intervention or surgery comprised eight articles, and sixteen articles did not compare SMVr and TEER. Therefore, a total of eight studies were included in this review (4–8, 11, 14, 15).

### 3.2 Study and demographic characteristic

This review included 6,224 patients from multiple studies. All studies were published in English and included full texts. They were all observational studies, with seven being retrospective cohort studies and one being a prospective cohort study. Three of the included studies were propensity-score-matched studies.

Among 6,224 subjects, 3,139 (50.43%) underwent SMVr, while 3,085 (49.57%) underwent TEER. These studies were published between 2013 and 2023, with data collected from 1999–2020. The average patient age across eight studies was 68.65 (±9.52) years for SMVr group and 69.16 (±12.23) years for TEER. Most of the subjects were male: 59.6% in SMVr group and 60.23% in TEER group. Both groups exhibited similar comorbid conditions, including atrial fibrillation, hypertension, diabetes mellitus, and chronic kidney disease. However, the TEER group had a more severe heart condition, with a higher proportion of subjects having MR grade 3 or higher (92.4% vs. 66.67%, *p* < 0.0001) and NHYA classification 3 or higher (73.95% vs. 63.50%, *p* = 0.003). The study characteristics are summarized in Table 1.

**Table 1:** Summarize of baseline characteristics of the subjects.

Characteristics	SMVr	TEER	*p* value
Age, years	68.65 ± 9.52 (*n* = 3,139)	69.16 ± 12.23 (*n* = 3,139)	0.07
Male %	1,876/3,139 (59.76%)	1,858/3,085 (60.23%)	0.703
MR Grade ≥3	170/255 (66.67%)	231/250 (92.4%)	<0.0001
NYHA III/IV	261/411 (63.50%)	264/357 (73.95%)	0.003
Atrial Fibrillation	1,453/3,139 (46.29%)	1,480/3,085 (47.97%)	0.19
Hypertension	2,343/3,026 (77.43%)	2,363/3,006 (78.61%)	0.269
Diabetes	1,127/3,026 (37,24%)	1,143/3,006 (38.02%)	0.529
CKD	171/2,524 (6.78%)	173/2,480 (6.98%)	0.771

### 3.3 Quality assessment

Table 2 shows the quality assessment result of eight included studies. The eight included studies were subjected to critical analysis and assessment of their biased risk using NOS, a critical appraisal tool for retrospective cohort studies. This study identified six high-quality articles and two fair-quality articles were identified in this study.

**Table 2:** Quality assessment of included studies using NOS for cohort studies.

Study	Selection				Comparability		Outcome			Total Score	Quality Judgement
	1	2	3	4	1	2	1	2	3		
De Bonis et al.	*	-	*	*	-	-	*	*	*	6	High
Conradi et al.	*	*	-	*	-	-	*	-	*	5	Fair
Taramaso et al.	*	*	-	*	-	-	*	-	*	5	Fair
Gyoten et al.	*	-	-	*	*	*	*	-	*	6	High
Ondrus et al.	*	-	*	*	*	-	*	*	*	7	High
Amabile et al.	*	*	*	*	*	*	*	*	*	9	High
Okuno et al.	*	*	*	*	*	*	*	*	*	9	High
Majmundar et al.	*	*	*	*	*	*	*	-	*	8	High

*, star system in NOS which will be calculated into the total score; -, counted as 0 in NOS. Description for Selection: 1 = Is the case definition adequate? 2 = Representativeness of the cases; 3 = Selection of controls; and 4 = Definition of controls. Description for second element is comparability of cases and controls based on the design or analysis and each study can have up to two stars. Outcome element contains: 1 = Assessment of outcome; 2 = Was follow-up long enough for outcomes to occur? and 3 = Adequacy of follow-up of cohorts.

### 3.4 In-Hospital mortality and procedural outcomes

**Figure 2:**
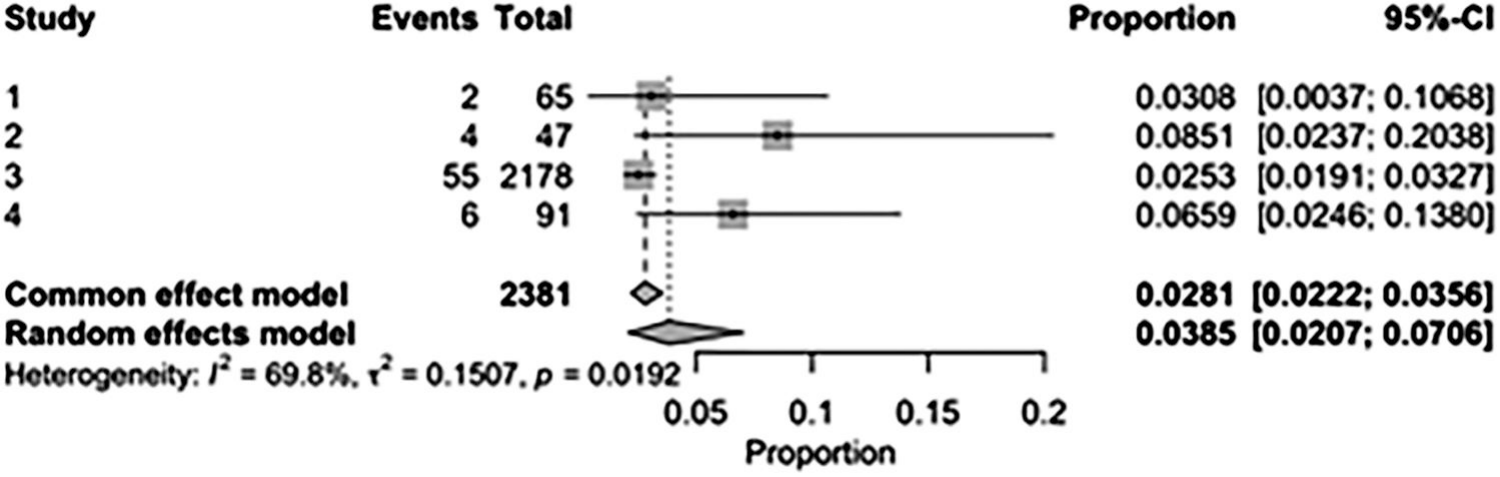
Forest plot for the pooled proportion of in-hospital mortality in the SMVr group.

**Figure 3:**
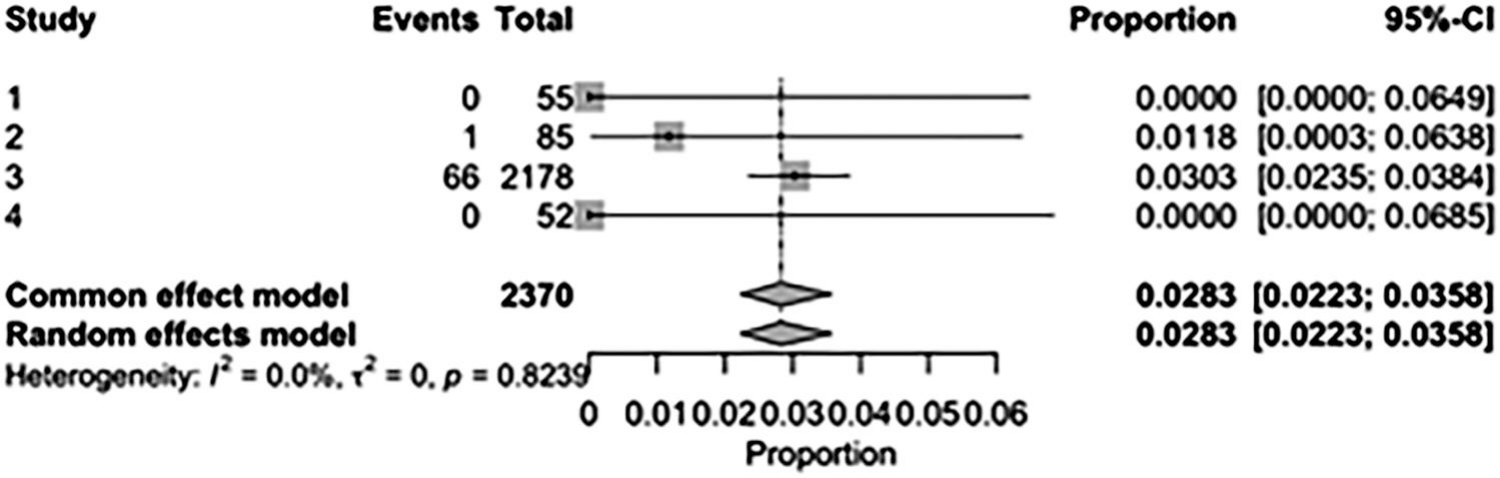
Forest plot for the pooled proportion of in-hospital mortality in the TEER group.

**Figure 4:**
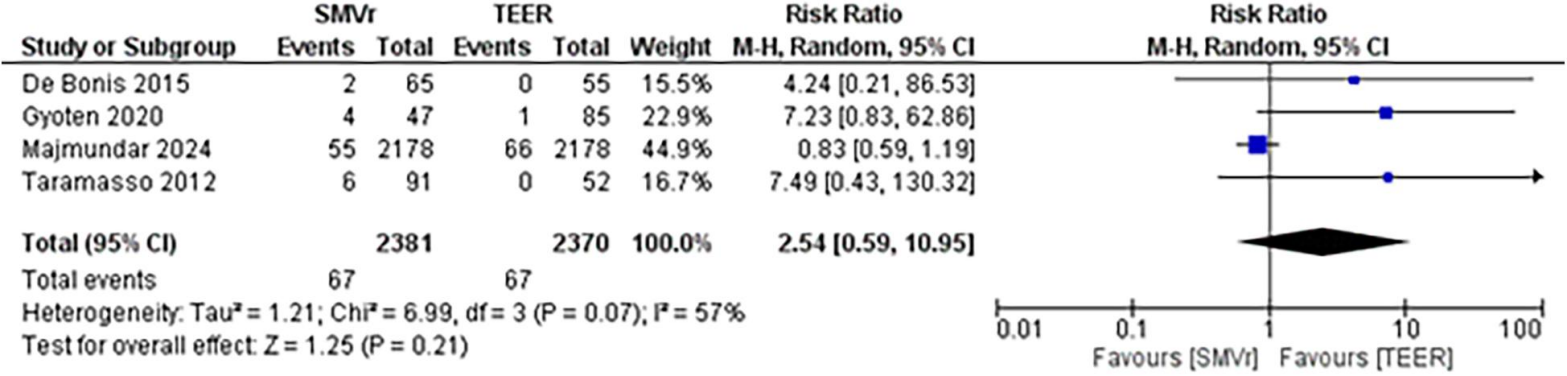
Forest plot for in-hospital mortality between SMVr and TEER group.

**Figure 5:**
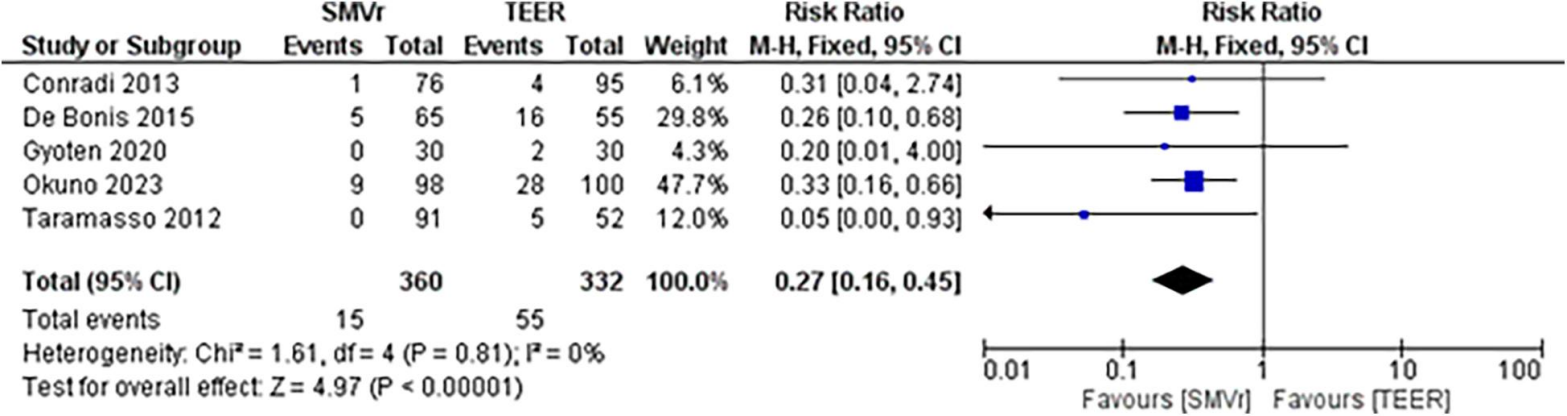
Forest plot for post-discharge residual MR between SMVr and TEER group.

**Figure 6:**
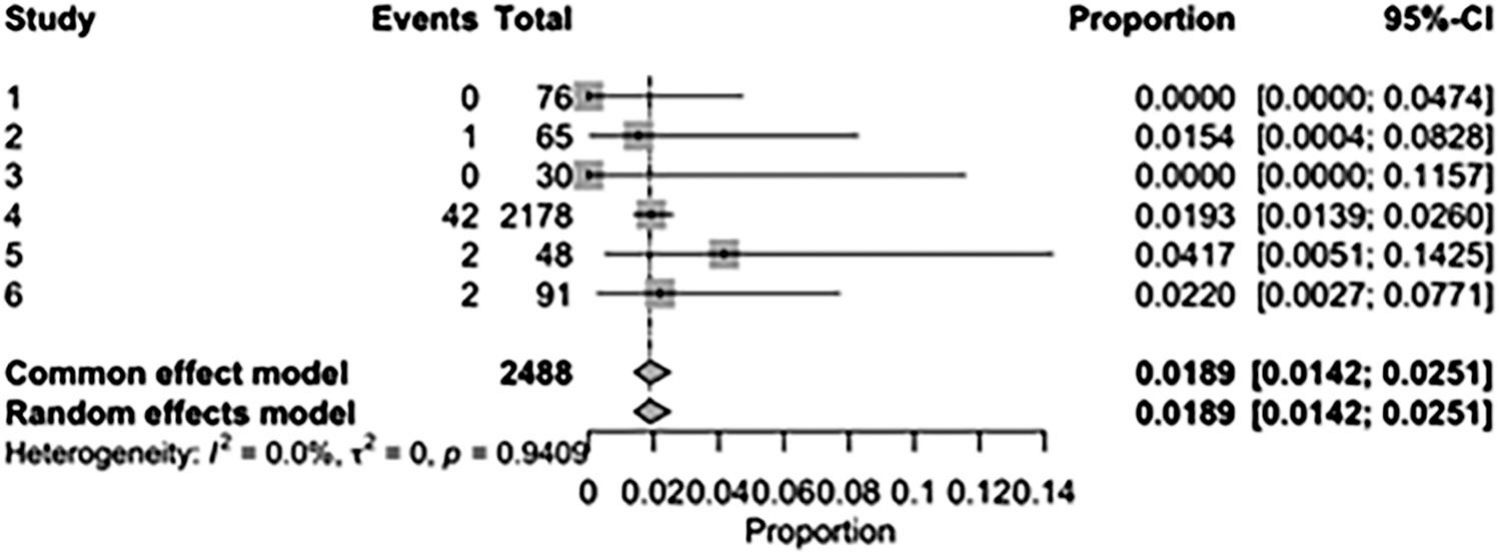
Forest plot for the pooled proportion of neurologic events in the SMVr group.

**Figure 7:**
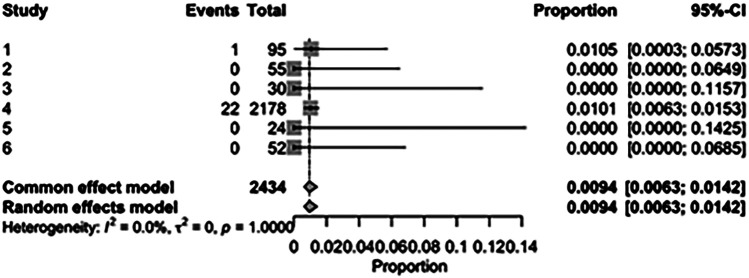
Forest plot for the pooled proportion of neurologic events in the TEER group.

**Figure 8:**
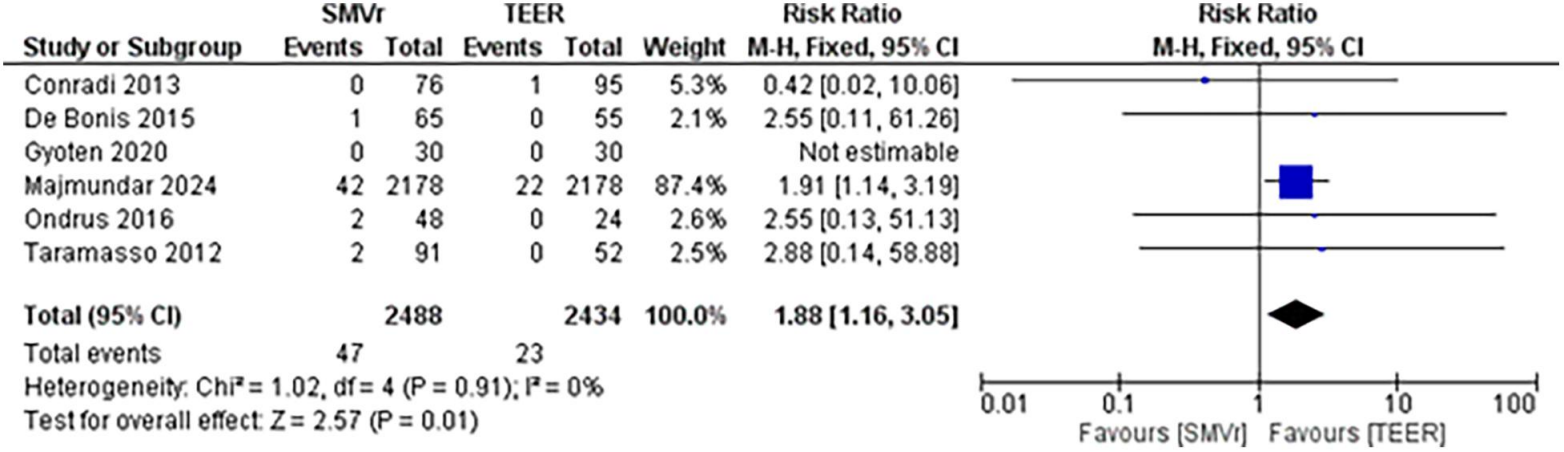
Forest plot for neurologic event between SMVr and TEER group.

**Figure 9:**
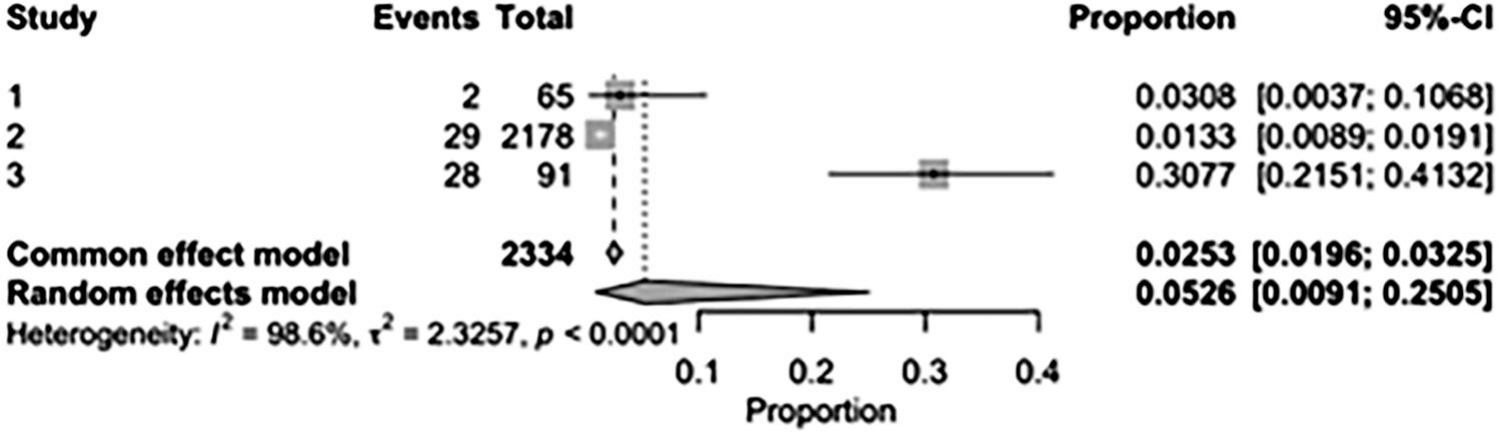
Forest plot for the pooled proportion of acute renal failure in the SMVr group.

**Figure 10:**
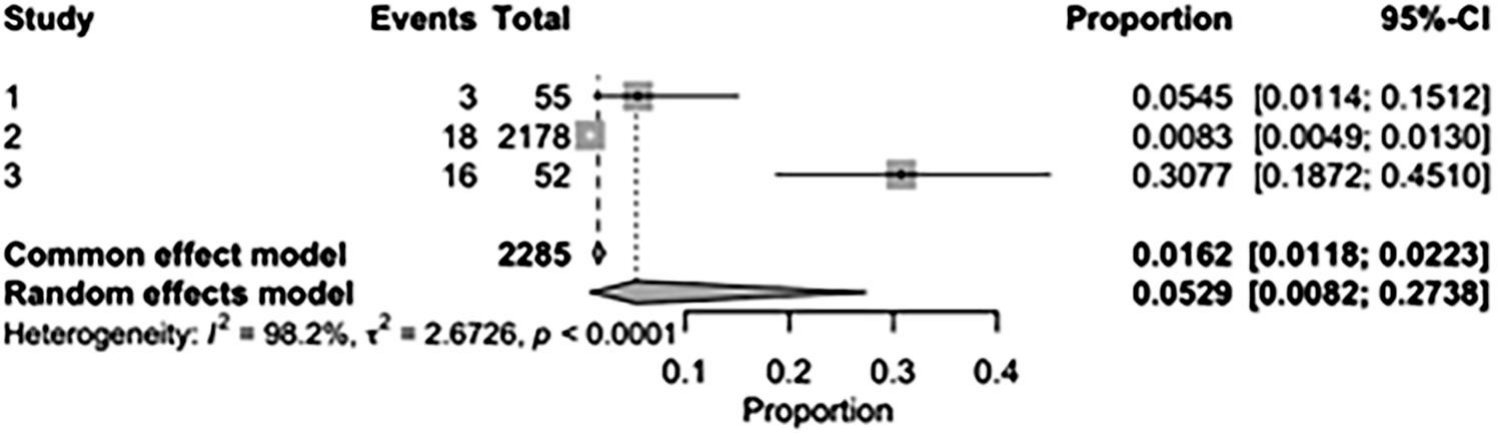
Forest plot for the pooled proportion of acute renal failure in the TEER group.

**Figure 11:**
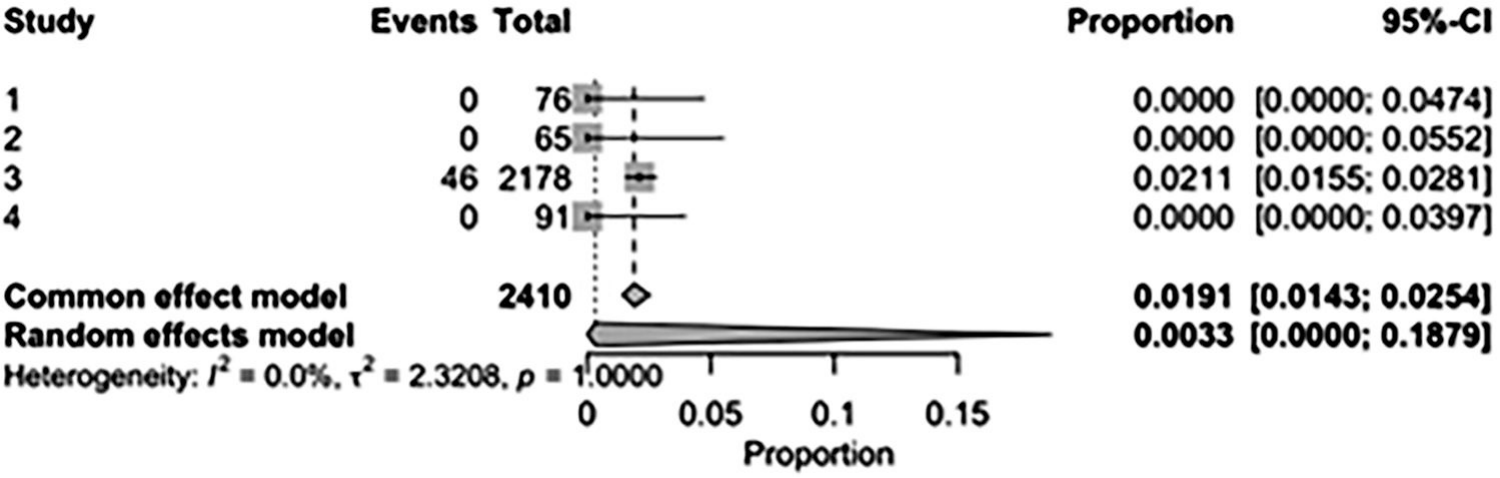
Forest plot for the pooled proportion of postoperative myocardial infarction in the SMVr group.

**Figure 12:**
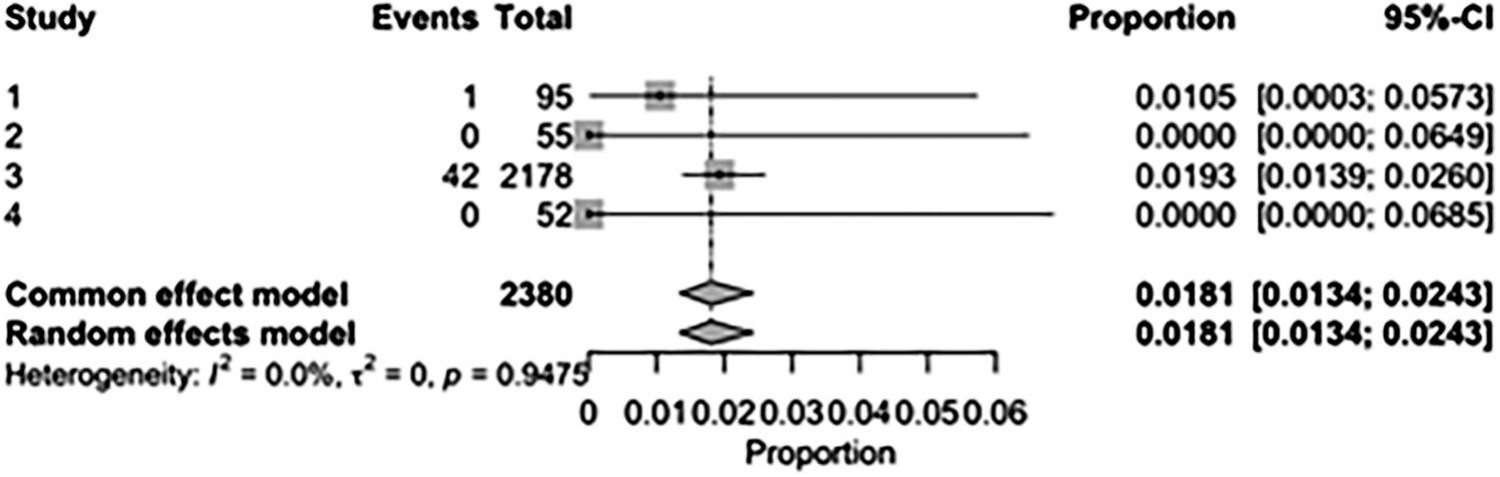
Forest plot for the pooled proportion of postoperative myocardial infarction in the TEER group.

**Figure 13:**
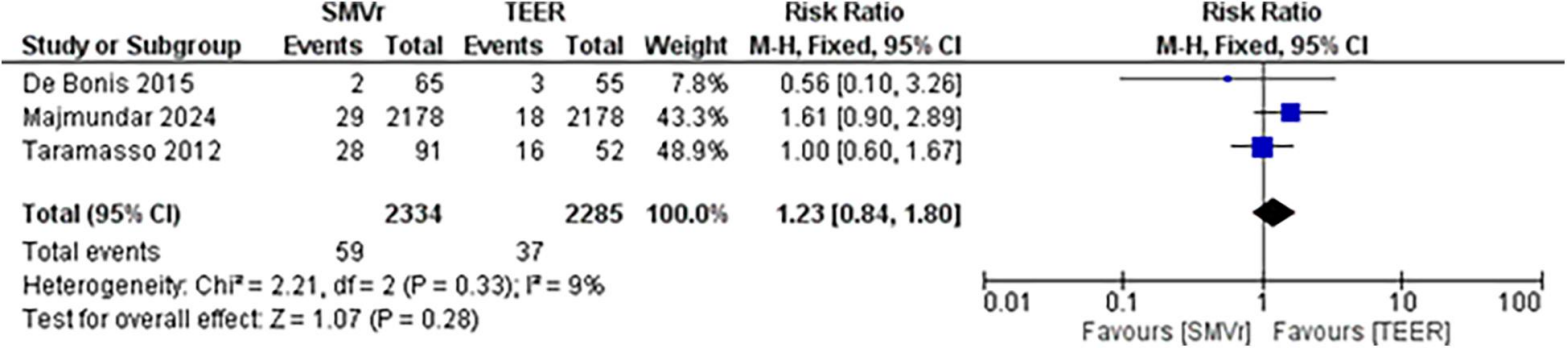
Forest plot for acute renal failure between SMVr and TEER group.

**Figure 14:**
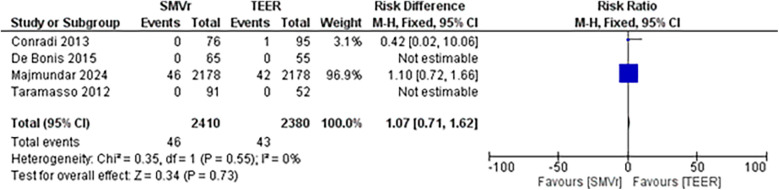
Forest plot for postoperative myocardial infarct between SMVr and TEER group.

The pooled incidence of in-hospital mortality was 3.85% [95% CI: 2.07%–7.06%; Figure 2] in the SMVr group and 2.83% [95% CI: 2.23%–3.58%; Figure 3] in the TEER group, with no statistically significant differences (RR = 2.54; 95% CI = 0.59–10.95; *p* = 0.21; I^2^ = 57%; Figure 4). The pooled data analysis of 692 patients from five studies suggests that patients who underwent SMVr are 73% less likely to experience post-discharge residual MR compared to those underwent TEER (RR = 0.27; 95% CI = 0.16–0.45; *p* < 0.01; I^2^ = 0%; Figure 5). The pooled incidence of neurologic event was found to be 1.89% [95% CI: 1.42%–2.51%; Figure 6] in the SMVr group and 0.94% [95% CI: 0.63%–1.42%; Figure 7] in the TEER group, with a significantly higher rate in the SMVr group (RR = 1.88; 95% CI = 1.16–3.05; *p* = 0.01; I^2^ = 0%; Figure 8). The pooled incidence of acute renal failure was 5.26% [95% CI: 0.91%–25.05%; Figure 9] in the SMVr group and 5.29% [95% CI: 0.82%–27.38%; Figure 10] in the TEER group. The incidence of postoperative myocardial infarction was 1.91% [95% CI: 1.43%–2.54%; Figure 11] in the SMVr group and 1.81% [95% CI: 1.34%–2.43%; Figure 12] in the TEER group. Both outcomes were higher in the SMVr group but did not reach statistical significance (acute renal failure: RR = 1.23; 95% CI = 0.84–1.80; *p* = 0.28; I^2^ = 9%; Figure 13; postoperative myocardial infarction: RR = 1.07; 95% CI = 0.71–1.62; *p* = 0.73; I^2^ = 0%; Figure 14).

### 3.5 Mid term outcome

#### 3.5.1 Mid term mortality

**Figure 15:**
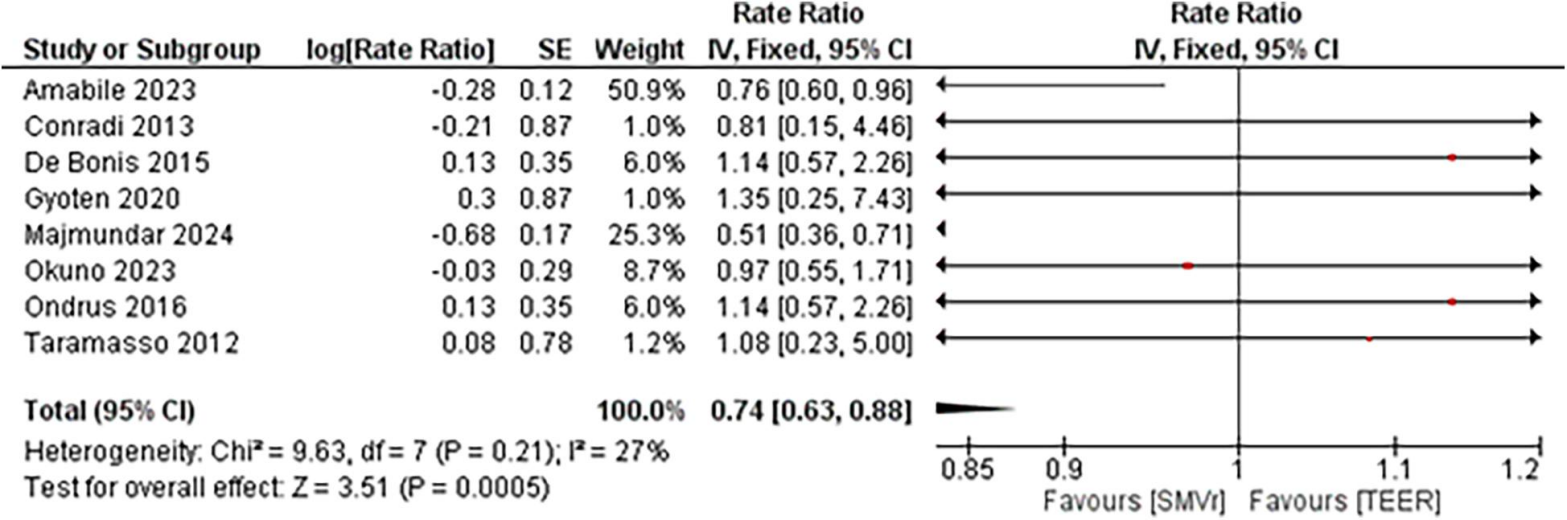
Forest plot for mid-term mortality between SMVr and TEER group.

Despite in-hospital mortality outcome analysis favoring the TEER strategy, during a total follow-up of 211,976 person-months, 253 deaths occurred in the SMVr group and 676 deaths in the TEER group. Pooled analysis demonstrated that SMVr was associated with a significantly lower mortality rate compared with TEER (Rate Ratio 0.74; 95% CI, 0.63–0.88; *p* < 0.001; I² = 27%; Figure 15). We included studies either with propensity score matching or without propensity score matching for the mid-term mortality analysis.

#### 3.5.2 Reintervention rate

**Figure 16:**
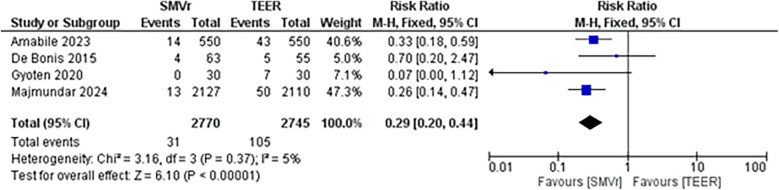
Forest plot for reintervention rate between SMVr and TEER group.

**Figure 17:**
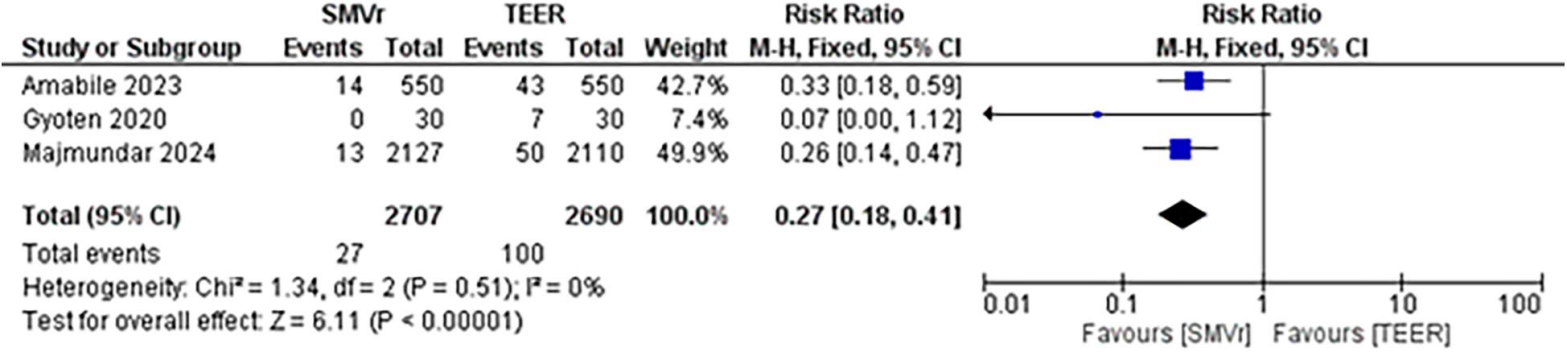
Forest plot with matched analysis for reintervention rate between SMVr and TEER group.

**Figure 18:**
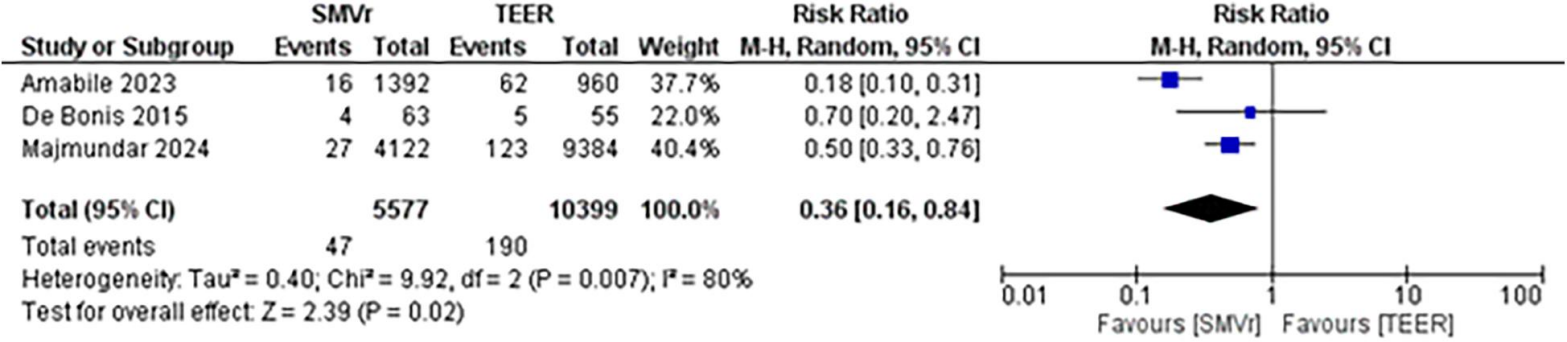
Forest plot with unmatched analysis for reintervention rate between SMVr and TEER group.

A pooled analysis of 5,515 patients from four studies found a statistically significant difference in the proportion of reintervention favoring SMVr strategies (RR = 0.29; 95% CI = 0.20–0.44; *p* < 0.001; Figure 16). The rates displayed low statistical heterogeneity with I^2^ = 5%. The analysis showed similar results when the studies were either divided into matched (RR = 0.27; 95% CI = 0.18–0.41; *p* = <0.001; I^2^ = 0%; Figure 17) and unmatched analysis (RR = 0.36; 95% CI = 0.16–0.84; *p* = 0.02; Figure 18). However, there was a substantial statistical heterogeneity in unmatched analysis (I^2^ = 80%).

#### 3.5.3 Recurrent MR and rehospitalization due to heart failure

**Figure 19:**
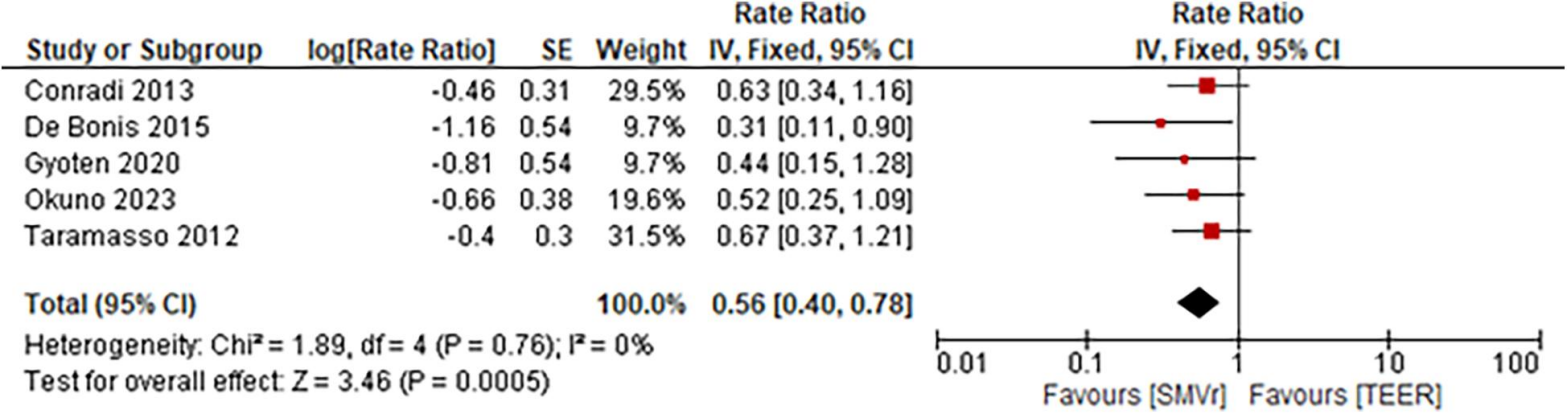
Forest plot for recurrent MR between SMVr and TEER group.

**Figure 20:**
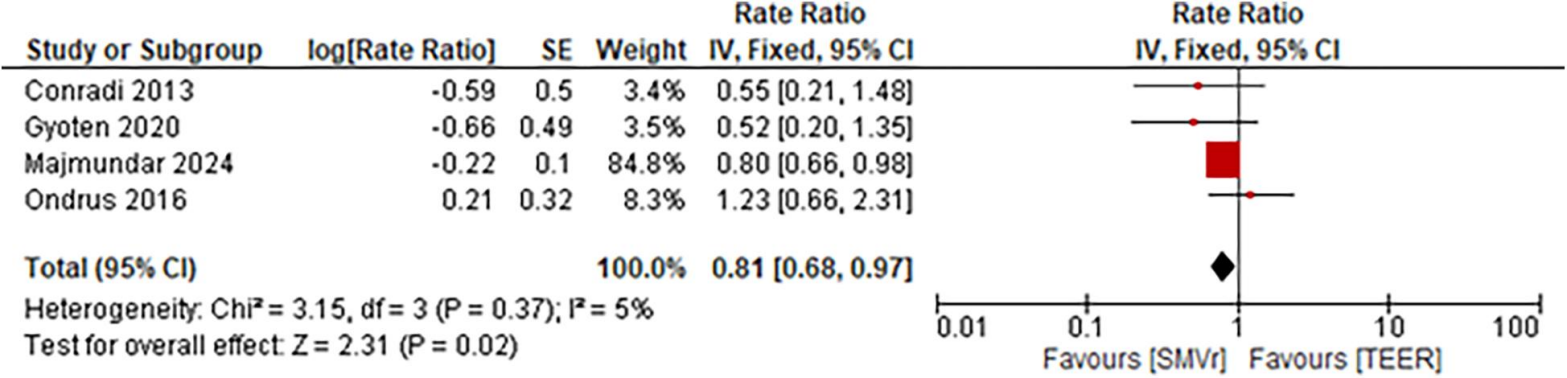
Forest plot for rehospitalization due to heart failure between SMVr and TEER group.

The incidence rate of recurrent MR during follow-up was significantly lower and favored the SMVr group (Rate Ratio = 0.56; 95% CI = 0.40–0.78; *p* = 0.0005; I^2^ = 0%; Figure 19). Similarly, the incidence rate of rehospitalization due to heart failure was significantly lower in the SMVr group (Rate Ratio = 0.81; 95% CI = 0.68–0.97; *p* = 0.02; I^2^ = 5%; Figure 20).

**Figure 21:**
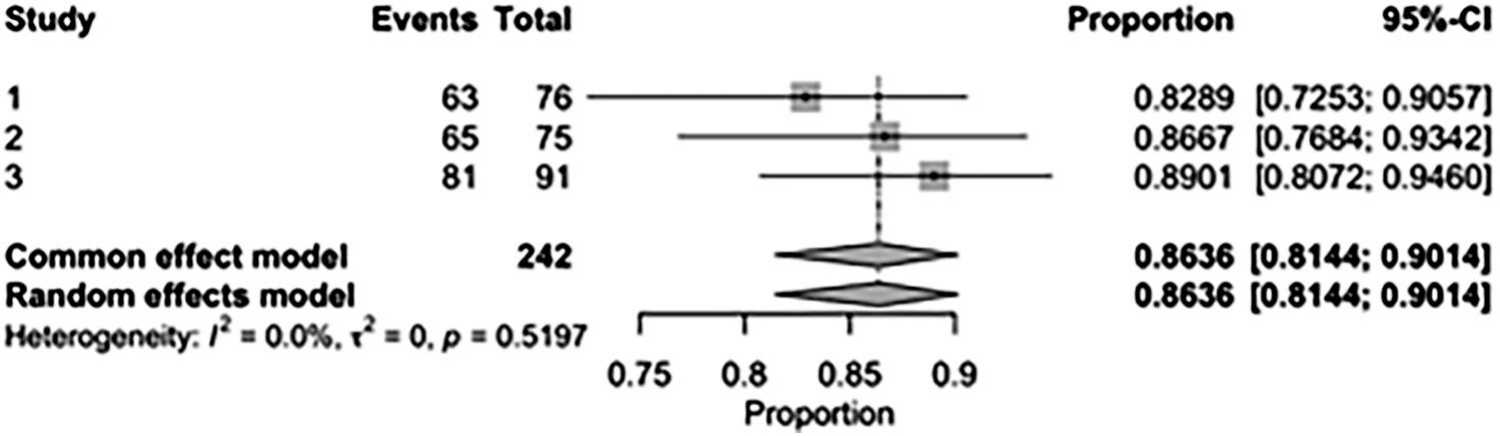
Forest plot for the pooled proportion of the improved functional status (NYHA) in the SMVr group.

**Figure 22:**
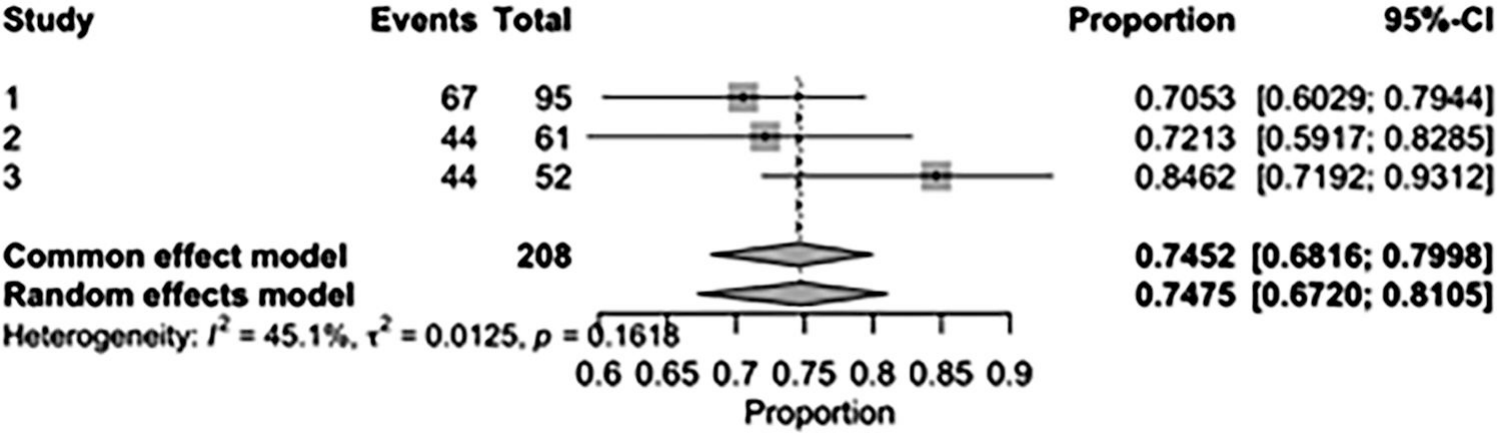
Forest plot for the pooled proportion of the improved functional status (NYHA) in the TEER group.

**Figure 23:**
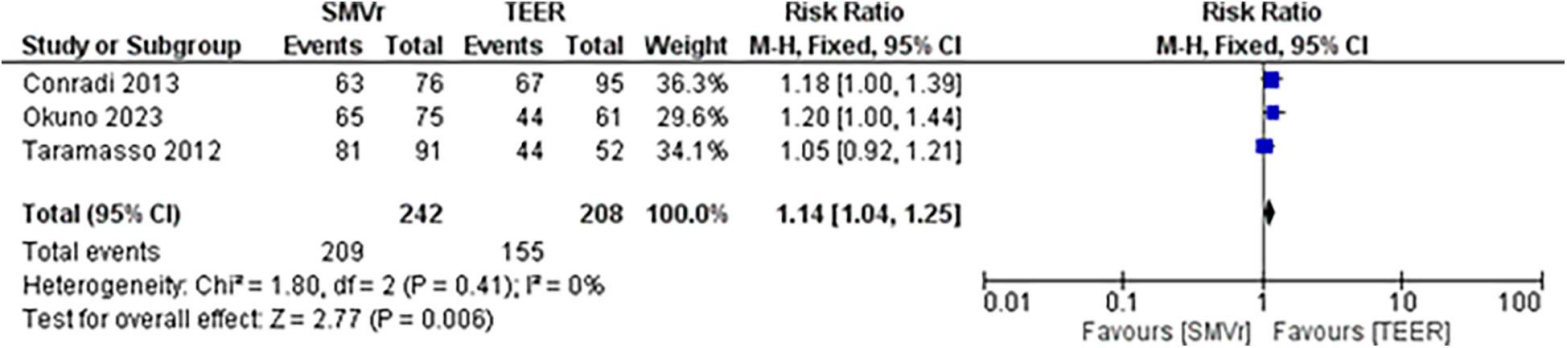
Forest plot for the improved functional status (NYHA) between SMVr and TEER group.

The pooled rate of improvement in functional status (NYHA) was 86.36% [95% CI: 81.44%–90.14%; Figure 21] in the SMVr group and 74.75% [95% CI: 67.20%–81.05%; Figure 22] in the TEER group. The pooled data analysis of 450 patients from three studies suggests that patients who underwent SMVr are more likely to experience this improvement (RR = 1.14; 95% CI = 1.04–1.25; *p* < 0.006, I^2^ = 0%; Figure 23) compared to those who underwent TEER.

## 4 Discussion

Several systematic reviews and meta-analyses compared the outcomes of surgical mitral valve repair (SMVr) and transcatheter edge-to-edge mitral valve repair (TEER) for mitral regurgitation. However, these studies did not specify the subject of secondary mitral regurgitation patients. Furthermore, no studies have directly compared the outcomes between SMVr and TEER in these subjects.

Our meta-analysis showed a significantly lower incidence of mid-term mortality, reintervention rate during the follow-up period, recurrent MR, and rehospitalization due to heart failure in the SMVr group compared to the TEER group. The in-hospital mortality rate for SMVr and TEER was comparable (3.85% vs. 2.83%), while the mid-term mortality outcome favored SMVr. However, there were moderate to substantial statistical heterogeneities in the results. These heterogeneities can result from several factors, including the baseline characteristics of each patient between two groups, the distribution and variances in treatment strategies across all centers, the facilities used for the treatments, the operator's skill, and the timing of surgical procedures.

Regarding risk factors and demographics, patients in the SMVr and TEER groups differ. The TEER patients were slightly older in terms of age and had a higher number of patients with NYHA class III or IV and MR grade 3 or higher than the SMVr patients. The higher logistic EuroSCORE values clearly indicate a greater mortality risk in TEER patients. Consequently, variations in the patient's initial conditions likely contribute to the disparities in clinical outcomes between these two groups. This consideration is crucial when evaluating postoperative clinical outcomes, especially mortality. However, it remains possible to compare the two groups to determine whether their approaches compete or complement each other and to identify and delineate the differences between these distinct populations. Despite the higher perioperative risk of mortality in TEER, it has demonstrated safety in the short-term outcomes. However, the tendencies of a lower 30-day mortality rate and mid-term mortality rate in SMVr group must be noted. The higher mortality in TEER group is mostly due to the patient's age and more complex comorbidities. There were several factors associated with mid-term mortality in TEER for overall mitral valve regurgitation, including age > 75 years old (15), NYHA class IV, anemia, previous aortic valve intervention, renal failure with serum creatinine ≥1.5 mg/dl, peripheral artery disease, left ventricular ejection fraction <30%, and severe tricuspid regurgitation (16). Polimeni et al. (17) reported that left ventricular end-diastolic volume index (LVEDVi) and NYHA class were independent predictors of rehospitalization for HF or cardiovascular death in patients undergoing TEER. In this study, NHYA class was significantly different between the two groups, which might be a consideration of the mortality outcome. Moreover, the lower MR grade in the SMVr group likely reflects that surgery was performed for other concomitant cardiac conditions—such as CABG for coronary artery disease—even when MR was only mild to moderate.

Other than comorbidities, treatment strategies used and procedural failure might increase the risk of mortality due to some reasons. First, experienced institutions and operators can enhance procedure outcomes (18, 19). In the context of mitral valve repair, a higher annual case volume for surgeons is associated with improved repair rates, greater freedom from reoperation, and enhanced survival outcomes (20). The more experienced the operator, the lower the procedure time and the complication rates (21). At the institutional level, there is a correlation between higher annual mitral valve surgery volume and improved repair rates, as well as a reduction in mortality. The association between better outcomes and procedures performed at high-volume hospitals may be attributed to factors such as operator and institutional experience, selective referral of lower-risk patients, and improved process of care management (22). The variation in institution and operator skills was not mentioned in the included studies. This factor could impact the outcomes and contribute to the substantial statistical heterogeneities in this result.

Second, the success of the procedure has an impact on the patient's outcome. One-year survival was significantly reduced in patients with MR 3+/4+ at discharge, which is commonly referred to as procedural failures (52.4%, *P* = 0.001 in comparison with MR ≤1+ and *P* = 0.02 in contrast with MR 2+, respectively) (16). Okuno et al. (23) reported that SMVr outcomes demonstrate a sustained reduction of mitral regurgitation (MR) in over 85% of patients throughout a two-year follow-up period. It is aligned with our results which confirm that patients undergoing TEER tend to experience a higher incidence of residual MR after discharge compared to those undergoing SMVr. This observation is also consistent with the initial findings of the EVEREST II Trial and remains consistent across various studies, despite TEER being a less invasive option (24, 25). SMVr provides the benefit of customized repair based on the patient's individual anatomy and condition, which may lead to a more successful MR correction when compared to the standardized method of TEER. Additionally, SMVr typically results in a more durable repair than TEER, reducing the risk of mortality and recurrent MR over time. This durability is often attributed to the use of sutures and other permanent fixation techniques to stabilize the repaired valve leaflets.

The result of this study revealed that the reintervention rate was lower in SMVr than in the TEER group, even after propensity score matching. This finding aligns with the results from the EVEREST II trial, which showed higher rates of MV reintervention in patients undergoing TEER compared to those undergoing SMVr (26). In patients with primary mitral regurgitation (MR), the mitral valve (MV) leaflets can be damaged either by myxomatous degeneration or rheumatic heart disease. In heart failure with reduced ejection fraction (HFrEF), a dilated left ventricle can also cause dilatation of the MV annulus. Both conditions result in the inability of the MV leaflets to fully close, leading to regurgitation. In surgical mitral valve repair (SMVr) for primary MR, the damaged part of the MV leaflet is excised, sutures are placed, and the chordae tendineae are rearranged. For HFrEF patients, annuloplasty with the insertion of a mitral valve ring is performed. However, transcatheter edge-to-edge mitral valve repair (TEER) does not employ these techniques; instead, it utilizes a clip to grasp and bring together the mitral valve leaflets. The limited effectiveness of TEER in correcting MR (55%) is concerning, especially considering its association with further disease progression and long-term adverse outcomes. This may partly explain why surgical intervention tends to yield better results in terms of the reintervention rate and mid-term mortality.

Furthermore, TEER was associated with a higher recurrence of MR during follow-up periods of more than six months, poorer functional outcomes, and higher rates of heart failure (HF) rehospitalization. TEER is widely recognized as less effective in reducing MR; however, it was developed under the expectation that a less effective therapy might be acceptable if it proved to be safer (11). The safety and the efficacy of the percutaneous edge-to-edge technique with the MitraClip were initially tested in the EVEREST I trial and subsequently compared with surgery in the randomized EVEREST II trial. The results of the randomized EVEREST II trial showed that in carefully selected patients, the MitraClip treatment is superior in safety, with an acceptable margin of decreased efficacy in reducing MR compared to surgery. However, it's important to note that most patients enrolled in the EVEREST trials had degenerative MR (7).

On the other hand, functional MR (FMR) is associated with a poor prognosis in HF patients with post-ischaemic or idiopathic dilated cardiomyopathy. Irreversible heart function impairment may impact outcomes. Patients with a dilated left ventricle (LVEDVi > 92 ml/m^2^) are at an increased risk of cardiovascular death or rehospitalization due to heart failure (17). Surgical repair of severe FMR in this setting has been demonstrated to improve symptoms and quality of life, leading to reverse LV remodeling in a significant proportion of the patients (7). Surgical patients exhibited a lower rate of recurrent MR during follow-up. The absence of a concomitant annuloplasty might possibly explain the higher recurrence rate of MR in the percutaneous approach despite the initial restoration of valve competence (8). For patients who survive the perioperative stage, a surgical approach to treating FMR in HFrEF appears to be superior to percutaneous edge-to-edge repair. However, recurrent MR has a strong impact on rehospitalization rates for HF (5).

Despite the benefits, our results show no statistically significant difference in the occurrence of postoperative myocardial infarction and acute renal failure between the two groups, which is consistent with other studies (4, 8, 24) that found no significant differences in these complications between the SMVr and TEER groups. Both outcomes are linked to ischemic time. Two out of the three studies mentioned did not involve concurrent CAD in the patient group studied (8, 11). Furthermore, the SMVr utilized in all studies is a simple annuloplasty without extra repair methods (7, 8, 11). These minimize the SMVr ischemic time, ensuring kidney and heart perfusion.

In addition, this study revealed a significant increase in neurological events among patients undergoing SMVr compared to TEER. The incidence of stroke postcardiac surgery might occur through various pathophysiological pathways, predominantly caused by embolism. Atrial fibrillation, physical manipulation of the heart and atherosclerotic aorta, usage of cardiopulmonary bypass (CPB), and carotid artery stenosis might contribute to emboli formation during cardiac surgery (27, 28). The usage of CPB in SMVr might increase the risk of particulate and gaseous emboli entering the systemic circulation during CPB (28), and the time of the rewarming process may be a source of cerebral injury (29). Meanwhile, prior studies (24, 30, 31) did not observe a significant difference in stroke incidence between the TEER and SMVr groups. The modern circuits of CPB are equipped with an arterial line filter to minimize the embolic load, and the use of a blood salvage system when transfusing suctioned blood hinders the entry of emboli (28). These findings highlight the need for additional research on variables that may affect the risk of postoperative complications, especially in SMVr patients. This task is essential for improving clinical understanding and post-surgical management strategies to better direct patient care.

### 4.1 Limitations and suggestions

This meta-analysis predominantly included high-quality retrospective observational studies, with only one prospective cohort study contributed to the pooled result. Two potentially relevant studies could not be retrieved in full text and therefore were not included in our analysis. This may have introduced a risk of publication bias and should be considered when interpreting the findings. However, three propensity-score-matched studies, two of which involved large populations, were included in the analysis. Concomitant surgeries and different types of SMR may have influenced the pooled outcome. Further research focusing on specific SMR subtypes is warranted, as recommended by the 2025 ESC Guidelines on Valvular Heart Disease (32).

## 5 Conclusions

Surgical mitral valve repair (SMVr) has shown superior mid-term outcomes compared to transcatheter edge-to-edge mitral valve repair (TEER), including lower mortality rates at one year and beyond, reduced reintervention rates, and improved functional status in patients with mitral regurgitation. While in-hospital mortality rate was similar between SMVr dan TEER, the latter was associated with a higher incidence of residual mitral regurgitation after discharge and rehospitalization, but a lower rate of neurological events, particularly strokes, in comparison to SMVr.

This systematic review underscores the significance of considering patient demographics, comorbidities, procedural success, and operator expertise when evaluating outcomes for SMVr and TEER. Further research is essential to understand the factors influencing postoperative complications, especially in SMVr patients, to improve post-surgical management strategies and optimize patient care.

## Data Availability

The original contributions presented in the study are included in the article/Supplementary Material, further inquiries can be directed to the corresponding author.
