# Supplementary material for: Comparative analysis of postoperative outcomes following surgical and transcatheter edge-to-edge mitral valve repair for secondary mitral regurgitation: a meta-analysis & systematic review
Source: Front Surg. 2025 Oct 9;12:1645272. doi: 10.3389/fsurg.2025.1645272 (PMC12546329; doi:10.3389/fsurg.2025.1645272)
Supplement: Supplementary file 1 [file Table1.docx]

Supplementary Material

**Supplementary Table 1**. Baseline characteristics of the subjects

| Author, Year/ Country | Study Period | Number of patients | |  | Age, years | |  | Male (%) | |  | Predicted mortality | | |
| --- | --- | --- | --- | --- | --- | --- | --- | --- | --- | --- | --- | --- | --- |
|  |  | **TMVR** | **SMVR** |  | **TMVR** | **SMVR** |  | **TMVR** | **SMVR** |  | **System** | **TMVR** | **SMVR** |
| Majmundar, 2023/ USA | 2016 - 2019 | 2.178 | 2.178 |  | 68.1±13.2 | 68±9.8 |  | 56.90 | 57.70 |  | - | - | - |
| Okuno, 2021/ Switzerland | 2005 - 2018 | 101 | 101 |  | 69.3 ± 12 | 69.6 ± 6.7 |  | 63.4 | 69.3 |  | EuroSCORE II % | 8.07 (4.33-11.62) | 5.19 (3.48-10.03) |
| Amabile, 2023/ USA | 2015 - 2020 | 550 | 550 |  | 72.6 ± 10.1 | 72.1 ± 9.1 |  | 66.55 | 64.73 |  | - | - | - |
| Gyoten, 2020/ Germany | 2013 - 2017 | 30 | 30 |  | 71 ± 8.2 | 71 ± 8.5 |  | 70 | 61 |  | LogEuroSCORE Mean ± SD | 29 ± 16 | 30 ± 24 |
| Ondrus, 2016/ Belgium |  | 24 | 48 |  | 75 ± 9 | 76 ± 4 |  | 75 | 56 |  | EuroSCORE II % | 18 ± 14 | 14 ± 11 |
| Taramasso, 2012/ Italy | 2000 - 2011 | 52 | 91 |  | 68.4 ± 9.2 | 64.9 ± 9.8 |  | 82.7 | 76.9 |  | LogEuroSCORE % | 21.9 ± 4.8 | 10.2 ± 7.4 |
| De Bonis, 2015/ Italy | 1999 - 2011 | 55 | 65 |  | 68.3 ± 9.17 | 63.2 ± 10.05 |  | 83.6 | 69.2 |  | LogEuroSCORE Median (IQR) | 18.8 (10.8–28.2) | 11 (9–13) |
| Conradi, 2013/ Germany | 2002 - 2010 | 95 | 76 |  | 72.4 ± 8.1 | 64.5 ± 11.4 |  | 64.2 | 44.7 |  | LogEuroSCORE I % | 33.7 ± 18.7 | 10.1 ± 8.7 |

**Supplementary Table 1 (cont.)**. Baseline characteristics of the subjects

| Author, Year/ Country | MR Grade ≥3 (%) | | NYHA III/IV (%) | | Atrial Fibrillation (%) | | Hypertension (%) | | Diabetes (%) | | CKD (%) | | |
| --- | --- | --- | --- | --- | --- | --- | --- | --- | --- | --- | --- | --- | --- |
|  | **TMVR** | **SMVR** | **TMVR** | **SMVR** | **TMVR** | **SMVR** | **TMVR** | **SMVR** | **TMVR** | **SMVR** | **TMVR** | **SMVR** |  |
| Majmundar, 2023/ USA | - | - | - | - | 45.00 | 45.00 | 79.60 | 79.30 | 26.20 | 26.50 | 2.50 | 3.10 |  |
| Okuno, 2021/ Switzerland | 79.2 | 43.6 | 63.4 | 67.3 | 44.50 | 44.50 | 66.3 | 73.3 | 24.8 | 32.7 | 54.5 | 51.5 |  |
| Amabile, 2023/ USA | - | - | -  97 | - | 33.7 | 33.7 | 82.73 | 82.36 | 87.27 | 87.64 | - | - |  |
| Gyoten, 2020/ Germany | 97 | 97 |  | 97 | 29.7 | 29.7 | 97 | 90 | 50 | 23 | 47 | 30 |  |
| Ondrus, 2016/ Belgium | 79 | 46 | 88 | 92 | 58.91 | 58.91 | - | - | - | - | 48 | 44 |  |
| Taramasso, 2012/ Italy | - | - | 85 | 67 | 58.73 | 58.73 | 9.6 | 6.6 | 26.9 | 9.9 | 57.7 | 17.6 |  |
| De Bonis, 2015/ Italy | - | - | 82 | 86 | 43 | 43 | - | - | - | - | - | - |  |
| Conradi, 2013/ Germany | 100 | 99 | 98 | 88 | 67 | 67 | 76.8 | 73.7 | 40 | 25 | 9.9 | 6.6 |  |
